# The impact of emotional valence on generalization gradients

**DOI:** 10.3758/s13423-023-02450-8

**Published:** 2024-01-16

**Authors:** José A. Alcalá, Celia Martínez-Tomás, Gonzalo P. Urcelay, José A. Hinojosa

**Affiliations:** 1 Departamento de Psicología, Universidad Rey Juan Carlos, Madrid, Spain; 2 Instituto Pluridisciplinar, Universidad Complutense de Madrid, Madrid, Spain; 3 Departamento de Psicología Experimental, Procesos Cognitivos y Logopedia, Universidad Complutense de Madrid, Madrid, Spain; 4 School of Psychology, University of Nottingham, Nottingham, UK; 5 Centro de Investigación Nebrija en Cognición (CINC), Universidad Nebrija, Madrid, Spain

**Keywords:** Generalization gradients, Emotion, Language, Valence, Predictive learning

## Abstract

Generalization enables individuals to respond to novel stimuli based on previous experiences. The degree to which organisms respond is determined by their physical resemblance to the original conditioned stimulus (CS+), with a stronger response elicited by more similar stimuli, resulting in similarity-based generalization gradients. Recent research showed that cognitive or conceptual dimensions also result in gradients similar to those observed with manipulations of physical dimensions. Such findings suggest that attributes beyond physical similarity play a role in shaping generalization gradients. However, despite its adaptive relevance for survival, there is no study exploring the effectiveness of affective dimensions in shaping generalization gradients. In two experiments (135 Spanish and 150 English participants, respectively), we used an online predictive learning task, in which different stimuli (words and Gabor patches) were paired with the presence – or absence – of a fictitious shock. After training, we assessed whether valence (i.e., hedonic experience) conveyed by words shape generalization gradients. In Experiment 1, the outcome expectancy decreased monotonically with variations in valence of Spanish words, mirroring the gradient obtained with the physical dimension (line orientation). In Experiment 2, conducted with English words, a similar gradient was observed when non-trained (i.e., generalization) words varied along the valence dimension, but not when words were of neutral valence. The consistency of these findings across two different languages strengthens the reliability and validity of the affective dimension as a determinant of generalization gradients. Furthermore, our data highlight the importance of considering the role of affective features in generalization responses, advancing the interplay between emotion, language, and learning.

**Supplementary Information:**

The online version contains supplementary material available at 10.3758/s13423-023-02450-8.

## Introduction

The ability to predict the most likely consequences of specific events facilitates adapting to changing environments. Hearing the sound of a fire alarm likely activates a flight response. However, it would pose a significant challenge to our daily lives if we exhibited a flight response each time that we hear the sound of an ice-cream truck. While the former predictive cue evokes the perilous prospect of danger (conditioned stimulus [CS+]), the latter signal lacks any connection to an adverse outcome. Throughout our lives, we encounter new sounds that we have never experienced before, and we ponder whether to flee or not. The response to this dilemma is largely determined by the resemblance of the novel sound to the sound produced by the fire alarm or by anticipating a delicious ice-cream. A higher degree of physical similarity elicits a stronger response, which gradually diminishes as the characteristics of the novel stimuli deviate from the CS+. This pattern of behavior, known as generalization gradients, is widely observed across species and physical properties of stimuli (Shepard, 1987).

Both non-human and human animals exhibit generalization gradients based on physical attributes of stimuli such as color hues (Lee et al., 2018), sound properties (Baron, 1973), or the orientation of a line (Gallaghar et al., 2020). This progressive decline in responding reveals that organisms perceived them as belonging to a dimension that varies along a continuum, for example, a line with 0° of orientation to a line with 90° orientation. If the CS+ was a horizontal line, the response to that stimulus would be at their maximum level and monotonically decline as the orientation of the line moves away from the horizontal inclination. However, if organisms perceive each novel stimuli as completely unrelated to the CS+, the resulting response lacks a systematic relationship with the varying characteristics of the stimulus dimension, leading to the absence of a response.

Critically, the generalization process also occurs at a categorical level. For instance, Dunsmoor and colleagues (2012) associated instances of a category (e.g., animals) with a shock, but not instances of a different category (e.g., objects). Participants generalized their fear response to novel instances of the category associated with fear (despite animals being clearly different between them at a physical level), establishing associations at the level of the categorical knowledge. Such generalization can be achieved using words as CSs – via their semantic or conceptual representation. For example, Mertens et al. (2021) conducted a fear conditioning experiment using words conveying different sizes. The results demonstrated that participants' expectancy of the outcome and physiological responses (skin conductance) varied along the continuum of size, mirroring to a large extent what happens when conditioning is conducted with stimuli differing in a physical dimension (Lissek et al., 2008). These findings aligned with other studies that highlight the role of conceptual features in modulating the generalization response. For instance, it has been shown that semantic similarity between words enhances the transfer of fear responses (Boyle et al., 2016). In bilingual individuals, fear can be transferred from a specific word trained in one language to the same word in their second language (e.g., from *cup* in English to *taza* [cup] in Spanish; Grégoire & Greening, 2020). In these examples, words were not treated as neutral concepts; instead, each word conveyed a *previous* semantic representation that contributed to the observed generalization response.

Word processing involves access to different sources of semantic representations, including the retrieval of affective features. Based on the prevalent view of emotions (Russell, 1980), these features mainly include the dimensions of valence (the hedonic tone) and arousal (the level of activation). Of note, valence is represented through a functional continuum that ranges from unpleasant or negative to pleasant or positive, thus conceptually mirroring to a large extent the continuum observed in a physical dimension. Valence is an inherent characteristic of both non-human and human experiences (Lyon & Kuchling, 2021). It plays a critical role in determining organismic responses, promoting approach behavior when encountering positive events, (e.g., a ripe banana), and eliciting avoidance behavior in the presence of negative events, (e.g., a decaying banana). Moreover, there is substantial evidence that valence modulates basic cognitive processes (see Tyng et al., 2017). In the context of human communication, words convey valence, and thus influence our communication with our peers (Tamir et al., 2016). Prior research has shown valence effects at several stages of word comprehension and production (e.g., Hinojosa et al., 2020; Lindquist, 2021; Satpute & Lindquist, 2021). This enables us to express messages that are interpreted as conveying a sense of safety, neutrality, or threat. However, despite the biological and psychological relevance of valence, to the best of our knowledge, there are no studies exploring whether valence has an impact on generalization gradients. To fill this gap, we conducted two experiments to investigate whether changes in valence can result in generalization gradients.

## Experiment 1

The primary objective of Experiment 1 was to investigate whether participants spontaneously utilized the affective representation conveyed by words as a dimension influencing their responses when confronted with untrained stimuli, specifically novel words in the context of predictive learning. If valence is indeed being used as a dimension mapping the representation of the affective features of words, it would be reasonable to expect a linear decrement in the participants' response that aligns with the difference in valence between the generalization stimuli (GS) and the CS+. On the contrary, if valence has no influence on the participants' responses, one would anticipate observing either an irregular pattern or a flat gradient in their responses.

A differential training was conducted in which a word at the extreme of the valence dimension (positive [8] or negative [2]; counterbalanced) was paired with an outcome (US; a fictitious shock) and a word at the other extreme was associated with the absence of outcome (CS-). After training, a generalization test was conducted presenting the two conditioned words intermixed with five novel words close to the five integers between 3 and 7 in terms of valence (i.e., 3, 4, 5, 6, and 7). We anticipated that valence would determine the generalization gradients, showing a linear decrease of predictive responses in the direction opposed to the valence of the word paired with the outcome.

Furthermore, we explored whether the gradient (if any) of the affective dimension was of similar shape to a gradient of a physical dimension. We used orientation lines with a set of Gabor stimuli, ranging from 0° to 90°. We conducted the same differential predictive training as with the words. That is, the stimulus with the line orientation at one extreme of the continuum (e.g., 0°) was paired with the presence of the outcome, and the stimulus in the other extreme (e.g., 90°) with the absence of the outcome. During generalization, five novel stimuli with 15° differences were tested. In both scenarios we presented five GS between the CS+ and the CS- (five Gabor and five words, respectively). We anticipated a linear gradient when using line orientation (Gallaghar et al., 2020); nonetheless, the key question was whether the gradient obtained with the valence dimension was similar to the gradient achieved with degrees (i.e., physical dimension). The design was fully within-subjects, so each participant experienced the task with Gabor and words in a counterbalanced order.

### Method

#### Participants

One hundred and thirty-five Spanish students from the first year of psychology at the Complutense University of Madrid (26 males, 109 females, none non-binary; mean age 19.52 years) were recruited online and compensated with course credit. The participants had no previous experience with the task. Each participant provided informed consent.

No specific power analysis to calculate the sample size was conducted. We used as reference the sample size used in learning experiments exploring generalization gradients conducted online (e.g., Lee et al., 2018; Lovibond et al., 2020), in which a final sample of approximately 50 participants is achieved per condition.^1^ The experiment was approved by the Ethics Committee at the University of Nottingham.

#### Apparatus and material

The task was programmed using the Gorilla Experiment Builder (Anwyl-Irvine et al., 2020), inspired by Lovibond et al. (2020).

**Fig. 1:**
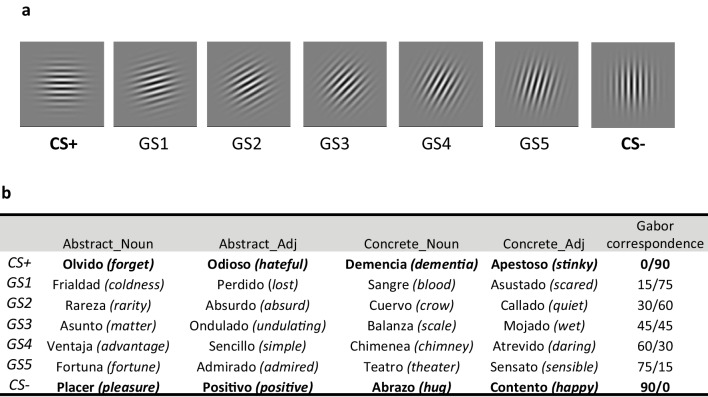
(**a**) Gabor stimuli, (**b**) word stimuli. *Note.* Figure 1a represents Gabor stimuli in which the CS+ was 0° and the CS- was 90° (counterbalanced). GS refers to generalization stimuli, and the numbers symbolize the distance with the CS+. Figure 1b represents the four subsets of lists used in the experiment and the correspondence with Gabor stimuli. Bold words are the trained words. In these examples, words of valence around 2 were used as CS+ and words with valence around 8 were used as CS- (counterbalanced)

Two different set of stimuli were used. First, a set of seven Gabor patches that differed in orientation line from 0° to 90° in steps of 15° (see Fig. 1a). Second, four different lists of words were selected via Emofinder (see Fig. 1b). The search was restricted to two databases in Spanish (Guasch et al., 2016; Hinojosa et al., 2016), which reported valence ratings in a 9-point scale (1, negative to 9, positive) and concreteness scores in a 7-point scale (1, abstract to 7, concrete). Valence features of words are typically based on subjective scores from participants on a 9-point Likert scale (1, negative to 9, positive; e.g., Hinojosa et al., 2016). We selected words around the integer’s ratings of valence from 2 to 8, ±0.40 points. The words had between six and eight letters and middle-to-high ratings of arousal (ranging between 3.5 and 7.5). Moreover, in order to rule out the possibility that the effects were driven by lexico-semantic factors that modulate the processing of emotional words –such as grammatical class or concreteness – (Hinojosa et al., 2020; Kousta et al., 2011; Palazova et al., 2011), we generated four lists of stimuli that included either concrete or abstract words that could be either nouns or adjectives (words were also matched on their frequency of use; Duchon et al., 2013). Concrete words had scores > 5, abstract words had scores < 3. Thus, four lists of stimuli were generated: concrete nouns, concrete adjectives, abstract nouns, and abstract adjectives. See Online Supplementary Material (OSM) for the detailed ratings of each word. There were no differences in terms of arousal, frequency, or valence across lists, (*p*s > .05).

### Procedure

The experiment was conducted online with the restriction that it had to be completed using a computer. After signing the consent form and providing demographic data, participants started the experiment. They conducted two versions of the same predictive learning task. The only difference between each task was the type of stimuli used (Gabor or words). The order in which participants conducted the task was counterbalanced (half of the participants started with the task using Gabor followed by words, and vice versa). Within each task, there was a discrimination training phase followed by a generalization test (e.g., training with words > Generalization test with words > Training with Gabor > Generalization test with Gabor). Before each phase, the same instructions appeared, with the only difference between both sets of instructions being the use of “symbol” or “word” to refer to the stimuli in each task (see the complete instructions in the OSM).

#### Training phase

The differential discrimination training comprised 12 presentations of the CS+ and 12 presentation of the CS-. The CS+ was probabilistically associated with the outcome according to a programmed contingency of 0.75: nine of the 12 CS+ trials were followed by the outcome. The CS- was deterministically associated with the absence of the outcome. Trials were presented in three blocks of four random presentations of each stimulus without any restriction. In the Gabor task two different stimuli with orientation lines of 0° or 90° were used as CSs (counterbalanced). In the Words task two different words with extreme values of valence were used (e.g., odioso *[hateful]* vs. positive *[positive]*; olvido *[forget]* vs. placer *[pleasure]*; apestoso *[stinky]* vs. contento *[happy]*; demencia *[dementia]* vs. abrazo *[hug]*); each pair corresponded to one of the four lists.

Each stimulus (Gabor or word) appeared on the upper center part of the screen. Below the stimulus appeared the question: *“The [symbol/word] above appears on the machine. What do you think will happen? SHOCK (press M) or NO SHOCK (press Z)”.* After participants responded, feedback showed *“Correct” or “Incorrect”* depending on their previous response**,** and the message: “*The previous [symbol/word] produced a SHOCK” or “The previous [symbol/word] did not produce a SHOCK”* depending on the programmed contingency. The feedback lasted 3 s on the screen. The intertrial interval (ITI) was 1.5 s. After each training phase, an expectancy test was conducted.

*Expectancy test.* Participants received the following instructions:

*For the next few trials, you will be shown some more [symbols/words], but you will NOT be shown feedback about whether a shock occurred. You should continue making predictions about whether you think a shock will occur. However, in this phase, you will be making your prediction on a scale ranging from “Definitely NO SHOCK” to “Definitely SHOCK.” Use your mouse to drag the slider along the scale to make your rating.*

Participants experienced seven stimuli in a random order without feedback about the outcomes. Note that this procedure does not result in extinction from testing several stimuli in the absence of outcome (see Lee et al., 2022). On each test trial, a stimulus appeared with the question: *“The [symbol/word] above appears on the machine. What is the likelihood of this stimulus leading to SHOCK?”* The horizontal scale ranged from 0 (“Certain No Shock”) to 100 (“Certain Shock”). Initially, the pointer of the slider appeared on the middle, and participants dragged the pointer to the left or right. They needed to click on the button “Confirm” to confirm their ratings. Participants were not allowed to continue to the next question unless they moved the slider and pressed “Confirm.” There was no time limit to respond and the ITI was 2 s. In the case of words, participant received the new words according to the type of training received (see Table 1).

**Table 1:** Lists of words for Valence Variable and Valence Fixed

**a:** Lists of words group Valence Variable				
	**Abstract noun**	**Abstract adj**	**Concrete noun**	**Concrete adj**
**CS+**	**betrayal**	**hopeless**	**mugger**	**killer**
GS1	tyranny	foolish	wounds	bloody
GS2	aversion	ominous	servant	wrinkled
GS3	protocol	annual	platform	curling
GS4	candor	rational	medicine	blonde
GS5	fantasy	creative	concert	bright
**CS-**	**kindness**	**lovable**	**laughte**	**wildlife**
**b:** Lists of words group Valence Fixed				
	**Abstract noun**	**Abstract adj**	**Concrete noun**	**Concrete adj**
**CS+**	**betrayal**	**hopeless**	**mugger**	**killer**
GS1	analogy	ongoing	headline	concrete
GS2	abstract	hypnotic	machine	staple
GS3	protocol	annual	platform	curling
GS4	standard	catchy	pulley	striped
GS5	tendency	nether	cauldron	vertical
**CS-**	**kindness**	**lovable**	**laughter**	**wildlife**

The trained words are shown in bold. Words of valence around 2 were used as conditioned stimulus (CS)+ and words with valence around 8 were used as CS- (counterbalanced across participants)

At the end of the experiment, we asked participants about their subjective commitment during the task with the following question:

*Well done! The experiment is over. Just one last question. Did you give your full attention to the experimental task (as opposed to sometimes doing other things like using your smartphone) while stimuli were being presented? Please, answer honestly, this question has no impact on your payment. There are two options below, “Yes” and “No”.*

After this question, the general rationale of the experiment was provided to the participants and they were debriefed.

#### Exclusion criteria

We used attentional, language, and learning criteria to ensure data quality. In the case of attentional checks, participants were removed if they declared not paying their full attention in the commitment question at the end of the experiment (see Alcalá et al., 2023 for similar criteria). Four participants declared not paying full attention. Additionally, we excluded participants who declared that Spanish was not their mother language (seven participants were excluded). Finally, participants were removed if they failed to learn the relationship between stimuli and outcomes during the training phase. They needed to demonstrate a higher proportion of responses to the CS+ compared to the CS- in the last block of training (see Lee et al., 2018, and Lovibond et al., 2020, for similar criteria). Participants who did not reach this level of discrimination in either task (Gabor and words) were removed from the analyses (11 participants were removed). After all these criteria, 106 participants were considered for analyses (21 males and 85 females; mean age 19.7 years).

#### Data analyses

During training, the proportion of responses predicting the shock for each stimulus was recorded. These data were used to apply the exclusion criteria at the end of training (see OSM for the trial-by-trial acquisition data).

During the expectancy phase, we first analyzed data using a factorial design with 2 (Dimension: Orientation vs. Valence) x 7 (Level: 1–7) x 2 (Order: Gabor First vs. Word First) analyses of variance (ANOVA). The first two dimensions were manipulated within subjects and the last manipulated between groups. After that, we evaluated the shape of the gradients considering the trend (linear, quadratic…) of the five GS. Moreover, we calculated the slope of the gradient as a measure of the steepness of the gradient. The rejection criterion was set at .05 for all statistical tests. Effect sizes and their confidence intervals are reported for tests relevant to the study hypothesis. Confidence intervals on partial-eta squares (95%) were computed using software available in Nelson (2016). When the assumption of sphericity was violated, the Huynh-Feldt correction was applied in the corresponding conditions.

﻿We further examined the data with mixed-design Bayesian ANOVAs. Specifically, we computed Bayes factor exclusions (*BF*_*excl*_) across matched-models that quantify the change from prior inclusion odds to posterior inclusion odds and can be interpreted as the evidence in the data for excluding one or several variables from the model fitting the data (see van den Bergh et al., 2020). For example, a *BF*_*exc*_ of 3 for the critical Dimension x Level interaction indicates that the data are three times more likely under models that do not include this interaction than under models with these specific predictors. Following the general guidelines for Bayesian analyses, we considered *BF* > 3 as substantial evidence in favor of the model tested (Jeffreys, 1961). These analyses were conducted with JASP 0.17 (JASP TEAM, 2023).

##### Supplementary analyses

In the OSM we provide analyses conducted with the subsets of lists of words. We firstly analyzed whether the valence of the word conditioned as CS+ (positive or negative) influenced the generalization gradients. Since we used a negative outcome, we may have found an asymmetry in the responses as a function of the valence of the conditioned word. Secondly, we analyzed concreteness (Abstract vs. Concrete) and the Type of word (Noun vs. Adjective) to rule out the possibility that these lexico-semantic factors modulated to some extent the responses across valence levels. These analyses are available in the OSM as they were not the main goal of the current experiment (but see *General discussion*).

### Results

**Fig. 2:**
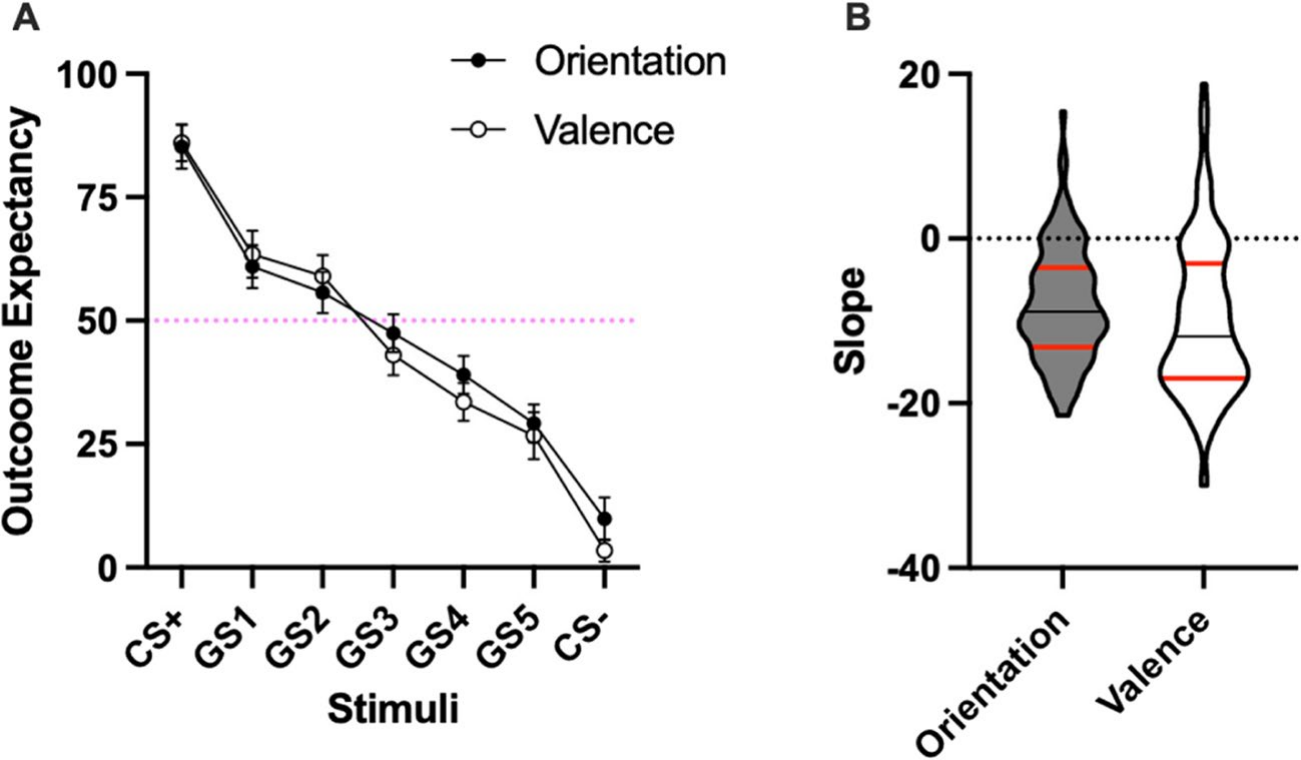
Generalization gradient. *Note.* Panel **A** represents the overall outcome expectancy for both stimuli dimensions. The CS+ was on the left part and the CS- on the right part of the figure. GS refers to generalization stimuli that were not presented during training. Numbers of GS refer to the proximity in terms of valence and orientation to the CS+ of each GS. GS1 was the closest to the CS+ and GS5 the furthest away. Error bars are the 95% confidence intervals (CIs). Panel **B** represents the violin plots of the slope considering the five GS. Black lines represent the medians and red lines the quartiles

As Fig. 2A shows, during test outcome expectancy for the CS+ was high for both dimensions (left part of the figure). Critically, the response progressively declined for stimuli further away from the CS+, until reaching their lowest level at the CS-. Moreover, the gradients of both dimensions seem very similar, without apparent differences between them. Repeated-measures ANOVA revealed that the triple interaction (Dimension x Level x Order) was not significant, *F*(6,624) = .95, *p* = .458, suggesting that Order did not modulate the critical interaction Dimension x Valence. There was a main effect of Level, *F*(3.17,316.58) = 323.45, *p* < .001, *η*^*2*^_*p*_ = .76, 95% CIs [.72, .79], but not of Dimension, *F*(1,104) = 2.36, *p* = .128, BF_exc_ = 5.27, nor a Level x Dimension interaction, *F*(3.04,316.58) = 2.15, *p* = .069, BF_exc_ = 6.32. Of note, Bayes factors provided reliable evidence for the absence of an interaction.^2^

Focusing on the five GS, there was a linear trend, *F*(1,105) = 232.78, *p < .001, η*^*2*^_*p*_ *= .*69 95% CIs [.59,.75], that was not modulated by Dimension, *F*(1,105) = 2.90, *p* = .090, suggesting that the linear trend was similar for both dimensions along the five GS. Furthermore, there were no differences in the slope between the two conditions, as shown in Fig. 2B, *t*(105) = 1.72, *p* = .09, *d* = 0.16, BF_01_ = 2.27. The Bayes factor only provided anecdotal evidence for the absence of differences. If anything, Fig. 2B suggests that there is a steeper slope for the valence dimension. Such differences may be driven by the facilitation observed when the CS+ was of negative valence, suggesting an easiness of conditioning between negative words and the putative aversive outcome (see OSM), compared to the use of a neutral dimension as the line orientation.

## Experiment 2

Experiment 1 revealed evidence supporting the use of valence as a dimension during the generalization test, showing a strikingly similar gradient to that of the physical dimension. Building upon these findings, Experiment 2 further investigated the role of valence as a dimension. In Experiment 1, GS words exhibited variations in valence, and the participants' responses closely aligned with those valence variations. In addition, participants were trained and tested in both stimuli sets, so it was possible that there was carry-over from the Gabor dimension to the valence dimension – despite absence of statistical evidence. In Experiment 2, a control group was introduced where the valence of the non-trained (GS) words remained constant during the test. In other words, the group Valence-Variable received the expectancy test with novel words varying along valence (similar to Experiment 1). Critically, the group Valence-Fixed were tested with five novel words of the same neutral valence (around integer five). Because the new words shared the same valence, we anticipated the absence of a gradient in this group, adjusting steadily to the neutral level of valence. In order to add generality to the findings of Experiment 1, we conducted this experiment with English speakers. If the same valence gradient is observed with English speakers, the main findings extend to other language and cultural contexts.

### Method

#### Participants

One hundred and fifty English speakers (58 males, 92 females; mean age 40.76 years) were recruited via Prolific and compensated with 1.2£. The participants had no previous experience with the task. Each participant provided informed consent. The same exclusion criteria as in Experiment 1 were applied. After the exclusion criteria, 137 participants were considered for analyses (72 in group Valence-Variable and 65 in group Valence-Fixed).

#### Apparatus, material, and procedure

The task was similar to the first experiment, except that only the condition with words was used. Four different lists of words in English were selected using several data bases. Valence ratings on a 9-point scale (1, negative to 9, positive) were extracted (Warriner et al., 2013). We selected words around the integer’s ratings of valence from 2 to 8, ± 0.40 points. Concreteness scores in a 5-point scale (1, very abstract to 5, very concrete) were used based on Brysbaert et al. (2014). Concrete words had scores > 3.5, abstract words had scores < 2.5. The words had between six and eight letters and middle-to-high ratings of arousal (ranging between 3.5 and 7.5). Frequency was extracted from Brysbaert and New (2009). Thus, the same type of lists as in Experiment 1 were generated (see Table 1). See OSM for the detailed ratings of each word in each parameter. As in Experiment 1, there were no differences in terms of arousal, frequency. or valence across lists, (*p*s > .05).

In the case of GS3, we used the same word for group Valence-Variable and Valence-Fixed. Note that the other GSs in the group Valence-Fixed were arbitrarily distributed. In fact, this number did not reflect the real distance with the CS+, and all GSs should be interpreted as GS3 in the Valence-Fixed group.

### Results

**Fig. 3:**
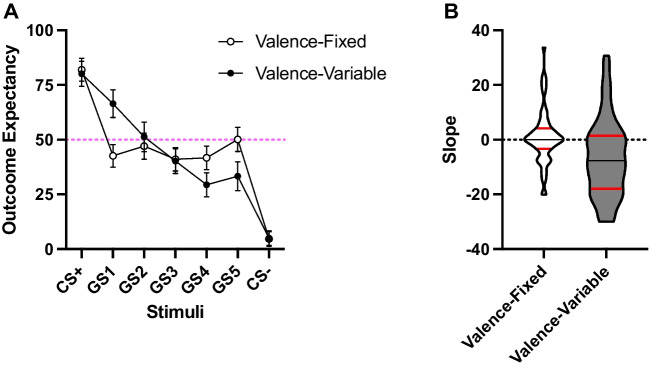
Expectancy test, Experiment 2. *Note.* Overall outcome expectancy for both stimuli dimensions. The CS+ is on the left part and the CS- on the right part of Fig. 3A. GS refers to generalization stimuli that were not presented during training. Number of GS refers to the proximity in terms of valence to the CS+ of each GS. GS1 was the closest to the CS+ and GS5 the furthest away. Error bars are the 95% confidence intervals (CIs). Panel B represents the violin plots of the slopes considering the five GS. Black lines represent the medians and red lines the quartiles

Figure 3A shows outcome expectancy during test. As expected, there were no apparent differences compared to the stimuli of similar valence across both groups, that is, to the CS+, CS-, and to the GS3 (the word with neutral valence in the group Valence-Variable). Importantly, there were differences in words not matched in valence. A mixed ANOVA revealed a Group x Level interaction, *F*(5.24,697.21) = 11.83, *p* < .001, *η*^*2*^_*p*_= .08, 95% CIs [.04,.12], BF_inc_ > 1000. Considering the five GS, the linear Level x Group interaction was significant, *F*(1,135) = 36.61, *p* < .001, *η*^*2*^_*p*_ = .21 [.10,.33] with a prominent linear trend in group Valence-Variable, *F*(1,71) = 46.32, *p* < .001, *η*^*2*^_*p*_ = .39 [.22,.53]. The quadratic component also was significant, *F*(1,71) = 12.82, *p* = .001, *η*^*2*^_*p*_ = .15 [.03,.30]. However, the linear trend was not significant in the group Valence-Fixed, *F*(1,64) = 1.11, *p* = .296. Analyses of the slope of the five GS showed differences between the two groups, *t*(135) = 3.56, *p* < .001*, d* = .61. In the case of the group Valence-Fixed, the slope was not different from zero, *t*(64) = 0.83, *p* = .405, *d* = 0.10, BF_01_ = 5.25, providing reliable support for the lack of a linear decrement.^3^

## General discussion

Two experiments were conducted to investigate whether valence influenced the formation of generalization gradients in a predictive learning scenario. Experiment 1 revealed a linear gradient, wherein changes in valence of words led to a gradual reduction in expectancy based on the proximity of the generalization stimuli (GS) to the conditioned stimulus (CS+). This decrement closely resembled the pattern observed when manipulating a physical dimension (line orientation). In Experiment 2, a control group was introduced, where the valence of words remained constant during the expectancy test. The results revealed a flat response in the Control group, in contrast to the well-defined linear gradient in a group in which words varied in valence during test. Hence, participants spontaneously applied the valence of words as a guide for their responses in the presence of non-trained (GS) words. Importantly, the presence of the linear gradient in both Spanish (Experiment 1) and English (Experiment 2) languages provides strong support for the generalizability of these findings.

Over-generalization of fear has been proposed as a fundamental mechanism underlying anxiety disorders and phobias (Lissek et al., 2008). Despite most studies investigating generalization gradients focusing on visual or auditory stimuli (i.e., physical dimensions), in recent years there is growing interest in the role played by conceptual information (e.g., Dunsmoor et al., 2012; Mertens et al., 2021). Interestingly, our findings demonstrated that the affective feature of valence also shaped the gradient. Mapping the mental representation of affective states has attracted attention from affective science, with a multidisciplinary perspective from cognitive psychology, psycholinguists, and cognitive neuroscience (see Hinojosa et al., 2020). Current data suggest an intricate interplay between learning, emotion, and language, likely reflecting the operation of domain-general learning processes (Heyes, 2019), regardless of the type of predictive dimension. In this regard, we observed that the representation of affective features may be a critical component when responding to non-trained (GS) stimuli, and this may be of major relevance not only as basic knowledge, but also in clinical settings.

Despite the rather abstract nature of the representation of the affective dimension, our observations consistently revealed a strong correspondence between valence and the orientation line, indicating that both dimensions influenced participants' responses in a similar manner. These findings go beyond what would be expected from a purely perceptual and objective resemblance between the conditioned stimulus and the generalization stimulus to account for the observed generalization gradient. On the contrary, our findings provide strong evidence supporting the notion that conceptual dimensions contribute to generalization responses (e.g., Mertens et al., 2021). These findings are consistent with Shepard’s (1987) principles of universal laws of generalization, which propose that the strength of a response decreases exponentially as the distance from the CS+ increases, and this decrement is contingent upon the psychological distance along the dimension in which the stimuli vary. In our study, participants' responses were indeed influenced by the valence-based distance.

Conditioning was conducted with words that varied along a subjective-normative dimension (unlike prototypical experiments using neutral stimuli). In line with this, we found an interaction between the valence of the CS+ during conditioning and the shape of the gradient (see OSM). That is, when conditioning occurred with a negative word as the CS+, the gradient was steeper, indicating sharper discrimination and consequently less generalization. This pattern is consistent with the idea that negative valence stimuli can better serve as predictors of aversive outcomes (e.g., Öhman & Mineka, 2001; but see Stussi et al., 2018). A logical prediction arising from this pattern is that if a positive outcome was used, the results might be the opposite, with a steeper gradient for positive valence words and greater reluctance to associate negative words with a positive outcome.

An interesting possibility to further explore the use of affective representation is to assess whether participants would still use the affective representation at test when training in different conditions (e.g., with single training [only training the CS+ in the absence of a CS-]), or testing stimuli further away from the CS during generalization. This would assess whether phenomena such as the peak-shift, previously observed when manipulating a physical dimension (e.g., Ahmed & Lovibond, 2019), can also be observed in the valence dimension.

Although we observed similar gradients in both the Spanish and the English experiments, it is important to note that differences in terms of the lexico-semantic components of words emerged between the two experiments. In Experiment 1, the pattern remained consistent regardless of both word type (noun vs. adjective) and word concreteness (concrete vs. abstract). However, in Experiment 2, we observed a modulatory effect of concreteness. In the English language, abstract words better fitted a linear gradient than concrete words, in which a flatter response was observed. Although potential differences between languages and concreteness are interesting, ﻿they are beyond the scope of the present series, which aimed to explore the general use of valence as a dimension in shaping the generalization gradient. Nonetheless, our findings are in line with prior observations indicating the existence of both cultural variations and universal aspects that underlie the representation of emotional features in words across languages (Jackson et al., 2019).

Our study is not free of some limitations. Semantic relationships could exist between some words that might have impacted the overall gradient. However, this possibility is unlikely since we used words conveying negative and positive emotions, which belong to different semantic domains. Also, the use of several lists in two languages might have mitigated this potential confound. Moreover, prior studies have shown that semantic coherence and emotional features have differential effects on word processing (Rossell & Nobre, 2004; Storbeck & Robinson, 2004).

Although the use of predictive learning scenarios is a valid approach for characterizing factors that determine the shape of generalization gradients (e.g., Lee et al., 2018; Vervliet et al., 2011)*,* future studies should test whether using aversive outcomes, such as a mild shock, result in similar findings. In the context of fear conditioning experiments, both explicit (i.e., prediction) and implicit (i.e., physiological responses) measures are conventionally employed. While there tends to be considerable overlap between both types of measures (e.g., Ahmed & Lovibond, 2019; Dunsmoor, et al., 2012; Mertens et al., 2021), it is noteworthy that such concordance is not consistently uniform. For instance, Grégoire and Greening (2020) found generalization of fear when evaluating self-reported fear and electrodermal activity, but not in the explicit measure of contingency. Hence, upcoming research may delve further into possible dissociations between explicit and implicit measures in the context of affective representation.

To sum up, affective representations shaped responses to non-trained (GS) words in a predictive learning scenario, following a pattern of responding tightly related to the valence of words. These results highlight the importance of considering affective features when studying the generalization response.

### Supplementary information

ESM 1 (DOCX 684 kb)

## Footnotes

1 In the case that the order of the task (Gabor first or Word first) interacted with some factors, we recruited more participants in order to have at least 50 participants in each condition. However, the order of the task did not interact, and consequently we pooled all data together.

2 In the Word dimension, we observed that the type of counterbalance (positive vs. negative) modulates the response across levels: *F*(4.58, 449.31) = 8.36, *p* < .001, *η*^*2*^_*p*_ = .08. This interaction is explored in the OSM. Concreteness and Type of word did not modulate the gradient

3 The valence of CS+ again modulated the shape of the gradient: *F*(5.24, 697.21) = 5.37, *p* < .001, *η*^*2*^_*p*_ = .04. This interaction is explored in the OSM. Unlike the first experiment, concreteness also modulated the response, see OSM.
