# Supplementary material for: The impact of emotional valence on generalization gradients
Source: Psychon Bull Rev. 2024 Jan 16;31(4):1670–9. doi: 10.3758/s13423-023-02450-8 (PMC11358170; doi:10.3758/s13423-023-02450-8)
Supplement: Supplementary file 1 — (DOCX 684 kb) [file 13423_2023_2450_MOESM1_ESM.docx]

**The Impact of Emotional Valence on Generalization Gradients**

José A. Alcalá^1,2,3^, Celia Martínez-Tomás^1,2^, Gonzalo P. Urcelay^4^, José A. Hinojosa^1,2,5^

^1^ Departamento de Psicología*,* Universidad Rey Juan Carlos, Spain

^2^ Instituto Pluridisciplinar, Universidad Complutense de Madrid, Madrid, Spain

^3^ Departamento de Psicología Experimental, Procesos Cognitivos y Logopedia, Universidad Complutense de Madrid, Madrid, Spain

^4^ School of Psychology, University of Nottingham, UK

^5^ Centro de Investigación Nebrija en Cognición (CINC), Universidad Nebrija, Madrid, Spain

**Experiment 1**

**1. Descriptive information about words**

**Supplementary Table 1:** Abstract noun list

| **Word** | **Level** | **Valence** | **Arousal** | **Concreteness** | **Log Frequency** |
| --- | --- | --- | --- | --- | --- |
| **Olvido (forget)** | 2 | 2.35 | 5.30 | 2.6 | 1.26 |
| Frialdad (coldness) | 3 | 3.33 | 4.81 | 2.35 | 0.76 |
| Rareza (rarity) | 4 | 4.27 | 4.35 | 2.5 | 0.47 |
| Asunto (matter) | 5 | 5.13 | 4.05 | 1.67 | 2.10 |
| Ventaja (advantage) | 6 | 6.36 | 4.15 | 2.9 | 1.61 |
| Fortuna (fortune) | 7 | 7.09 | 5.7 | 2.62 | 1.69 |
| **Placer (pleasure)** | 8 | 8.39 | 6.05 | 2.85 | 1.64 |

*Note. Words in bold were used as CSs during discrimination, the rest of the stimuli were Generalization Stimuli tested during test. All seven stimuli were tested during test.*

**Supplementary Table 2:** Concrete noun list

| **Word** | **Level** | **Valence** | **Arousal** | **Concreteness** | | **Log Frequency** | |
| --- | --- | --- | --- | --- | --- | --- | --- |
| **Demencia(dementia)** | **2** | **2.17** | **5.63** | | **5** | | **0.7** |
| Sangre (blood) | 3 | 2.95 | 7.18 | | 6.15 | | 2.05 |
| Cuervo (crow) | 4 | 4.19 | 5.14 | | 6.65 | | 0.83 |
| Balanza (scale) | 5 | 5.25 | 3.86 | | 6.05 | | 0.91 |
| Chimenea (chimney) | 6 | 5.9 | 3.8 | | 6.43 | | 0.96 |
| Teatro (theater) | 7 | 7.27 | 5.73 | | 5.73 | | 2.06 |
| **Abrazo (hug)** | **8** | **8.14** | **5.83** | | **5.86** | | **0.84** |

*Note. Words in bold were used as CSs during discrimination, the rest of the stimuli were Generalization Stimuli tested during test. All seven stimuli were tested during test.*

**Supplementary Table 3:** Abstract adjective list

| **Word** | **Level** | **Valence** | **Arousal** | **Concreteness** | **Log Frequency** |
| --- | --- | --- | --- | --- | --- |
| **Odioso (hateful)** | 2 | 1.63 | 6.13 | 3.83 | 0.49 |
| Perdido (lost) | 3 | 2.7 | 6.29 | 3.6 | 1.90 |
| Absurdo (absurd) | 4 | 4.05 | 4.23 | 3.23 | 1.23 |
| Ondulado (undulating) | 5 | 5.14 | 3.52 | 3.85 | 0.34 |
| Sencillo (simple) | 6 | 5.9 | 4.33 | 3.78 | 1.66 |
| Admirado (admired) | 7 | 6.95 | 6.68 | 3.74 | 0.78 |
| **Positivo (positive)** | 8 | 7.73 | 5.43 | 3.4 | 1.67 |

*Note. Words in bold were used as CSs during discrimination, the rest of the stimuli were Generalization Stimuli tested during test. All seven stimuli were tested during test.*

**Supplementary Table 4:** Concrete adjective list

| **Word** | **Level** | **Valence** | **Arousal** | **Concreteness** | **Log Frequency** |
| --- | --- | --- | --- | --- | --- |
| **Apestoso (stinky)** | 2 | 1.80 | 5.23 | 5.09 | 0.09 |
| Asustado (scared) | 3 | 2.70 | 6.10 | 5.43 | 0.87 |
| Callado (quiet) | 4 | 4.05 | 3.80 | 5.91 | 0.88 |
| Mojado (wet) | 5 | 4.67 | 5.27 | 5.67 | 0.63 |
| Atrevido (daring) | 6 | 6.27 | 6.09 | 5.17 | 0.98 |
| Sensato (sensible) | 7 | 7.00 | 4.47 | 5.05 | 0.76 |
| **Contento (happy)** | 8 | 8.10 | 6.70 | 5.40 | 1.30 |

*Note. Words in bold were used as CSs during discrimination, the rest of the stimuli were Generalization Stimuli tested during test. All seven stimuli were tested during test.*

**2. Training Instructions**

*We would like you to imagine that you have come across a strange machine. It appears to have a display on it, as well as a sign that says “WARNING: this machine gives electric shocks!! When you see warning [symbols/words] like this ______, do NOT touch!”. You will be in danger of being shocked.*

*Unfortunately, the area of the label that shows the warning symbols has been scratched off, so you do not know which symbols stimulus danger. Your job is to work out what kinds of symbols on the machine stimulus shock.*

*During the task you will be presented with different [symbols/words]. You will then make a prediction about whether you think a shock will occur. In the first part of the experiment, you will receive feedback for your predictions about whether a shock occurred or not.In the second part of the experiment, you will continue to make predictions and judgments. You will receive further instructions at the beginning of each phase.*

*In the first part, you will be able to predict which [symbols/words]lead to shock. We will present the same [symbols/words]to you MULTIPLE times.*

*Each time a [symbols/words] appeared on the machine, the next question will appear:*

*“The [symbols/words] above appeared on the machine. What do you think will happen?*

*Press* ***“m”*** *if you think a SHOCK will occur, or* ***“z”*** *if you think NO shock will occur.*

**3. Analyses of training**

A repeated measures 2 (Dimension: Valence vs Orientation) x 2 (CS: CS+ vs CS-) ANOVA conducted in the last training trial only revealed the expected effect of CS, *F*(1,105) = 1283, *p* < .001, *η^2^_p_*= .92, 95% CI [.90, .94], largest *F* for the interaction Dimension x CS, *F*(1,105) = 2.65, *p* = .106, suggesting that the discrimination was successful without differences between dimensions.

**Supplementary Figure 1:** Training of Experiment 1

*Note. Error bars are the 95% CIs.*

**4. Supplementary Analyses during expectancy test:**

*CS+ Valence:*

A repeated measures 2 (CS+Valence: Positive vs. Negative) x 7 (Level: 1-7) revealed an interaction *F*(4.58, 449.31) = 8.36, *p* < .001, *η^2^_p_*= .08, 95% CI [.03, .12]. Further analyses of the CS+Valence x Level showed that there was differences in the ratings to the CS+, with higher outcome expectancy in the case of negative conditioning CS+, *t*(104) = 2.05, *p* = .043, *d* = 0.40, but no in the case of the CS-, *t*(104) = 0.23, *p* = .812. Additionally, we evaluated the broadening of the gradient with the slope of the 5GS. As Figure S2B suggests, the slope for the negative conditioning was more pronounced, revealing a sharper gradient compared to the positive conditioning, *t*(104) = 4.52, *p* < .001, *d* = 0.88. In both cases, the slope was different from zero, smallest when the conditioning was conducted with positive valence, *t*(55) = 4.60, *p* < .001.

**Supplementary Figure 2**: Expectancy Test as a function of CS+Valence

*Note. Panel A represents the expectancy test when the CS+ has negative valence (black symbols) and positive valence (white symbols). Panel B represents the violin plot of the slope of the 5GS based on the valence of the CS+. Black lines represent the median and red lines the quartiles. Error bars are the 95% CIs.*

*Words Attributes:*

A repeated measures 2 (Type: Noun vs Adjetive) x 2 (Concreteness: Abstract vs Concrete) x 7 (Level: 1-7) did not reveal any significant interaction, either TypeWord x Level (Figure S3A), *F*(4.58, 449.31) = 0.74, *p* = .582 [BF_exc_ =59.38 ], or Level x Concreteness (Figure S3B), *F*(4.58, 449.31) = 1.16, *p* = .327 [BF_exc_= 37.28].

**Supplementary Figure 3**: Expectancy Test Words Attributes


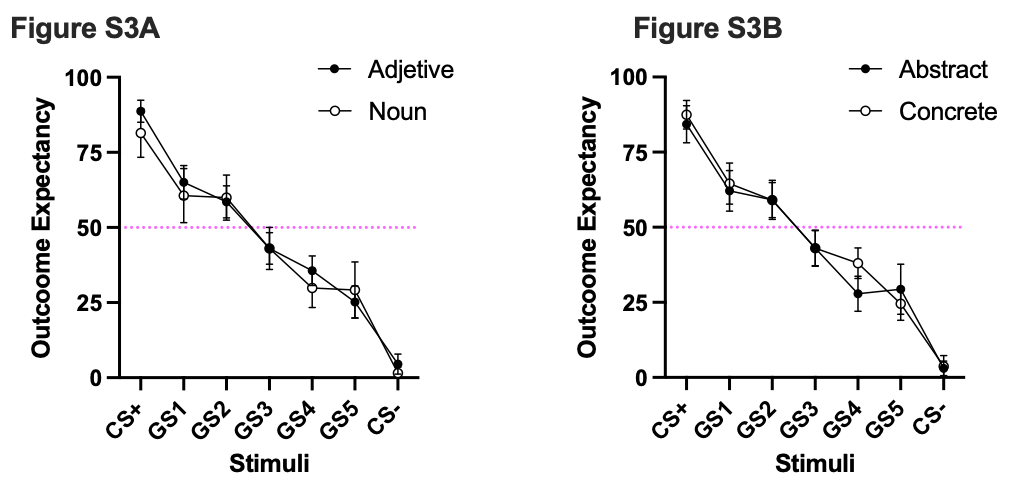


*Note. Panel A represents overall outcome expectancy for Adjectives and Noun lists. Panel B to Abstract vs. Concrete lists of words. Error bars are the 95% CIs.*

**Experiment 2**

**1. Descriptive information about words**

**Supplementary Table 5:** Abstract Noun Valence-Variable

| **Word** | **Level** | **Valence** | **Arousal** | **Concreteness** | **Log Frequency** |
| --- | --- | --- | --- | --- | --- |
| **betrayal** | **2** | **2.20** | **4.52** | **1.76** | **2.33** |
| tyranny | 3 | 3.19 | 5.45 | 1.68 | 1.91 |
| aversion | 4 | 4 | 5.14 | 1.85 | 1.83 |
| protocol | 5 | 5.10 | 3.83 | 1.97 | 2.58 |
| candor | 6 | 6.14 | 3.90 | 1.96 | 1.71 |
| fantasy | 7 | 7.15 | 6.18 | 1.59 | 2.91 |
| **kindness** | **8** | **7.65** | **3.96** | **1.82** | **2.66** |

*Note. Words in bold were used as CSs during discrimination, the rest of the stimuli were Generalization Stimuli tested during test. All seven stimuli were tested during test.*

**Supplementary Table 6**: Abstract Noun Valence-Fixed

| **Word** | **Level** | **Valence** | **Arousal** | **Concreteness** | **Log Frequency** |
| --- | --- | --- | --- | --- | --- |
| **betrayal** | **2** | **2.20** | **4.52** | **1.76** | **2.33** |
| analogy | 3 | 5.24 | 3.95 | 1.61 | 1.77 |
| abstract | 4 | 5.24 | 3.96 | 1.45 | 2.05 |
| protocol | 5 | 5.10 | 3.83 | 1.97 | 2.58 |
| standard | 6 | 4.76 | 3.61 | 1.85 | 2.56 |
| tendency | 7 | 4.85 | 3.72 | 1.83 | 2.14 |
| **kindness** | **8** | **7.65** | **3.96** | **1.82** | **2.66** |

*Note. Words in bold were used as CSs during discrimination, the rest of the stimuli were Generalization Stimuli tested during test. All seven stimuli were tested during test.*

**Supplementary Table 7**: Abstract Adjective Valence-Variable

| **Word** | **Level** | **Valence** | **Arousal** | **Concreteness** | **Log Frequency** |
| --- | --- | --- | --- | --- | --- |
| **hopeless** | **2** | **2.20** | **4.52** | **1.59** | **2.66** |
| foolish | 3 | 3 | 4.57 | 1.84 | 2.95 |
| ominous | 4 | 3.95 | 5.47 | 1.93 | 1.83 |
| annual | 5 | 5.19 | 3.70 | 1.78 | 2.56 |
| rational | 6 | 5.85 | 4.26 | 1.62 | 2.40 |
| creative | 7 | 7.06 | 4.86 | 1.93 | 2.73 |
| **lovable** | **8** | **8.26** | **5.41** | **1.97** | **1.96** |

*Note. Words in bold were used as CSs during discrimination, the rest of the stimuli were Generalization Stimuli tested during test. All seven stimuli were tested during test.*

**Supplementary Table 8**: Abstract Adjective Valence-Fixed

| **Word** | **Level** | **Valence** | **Arousal** | **Concreteness** | **Log Frequency** |
| --- | --- | --- | --- | --- | --- |
| **hopeless** | **2** | **2.20** | **4.52** | **1.59** | **2.66** |
| ongoing | 3 | 4.79 | 4.24 | 1.88 | 2.05 |
| hypnotic | 4 | 5.25 | 3.73 | 1.96 | 1.83 |
| annual | 5 | 5.19 | 3.70 | 1.78 | 2.56 |
| catchy | 6 | 5.22 | 4 | 1.79 | 2.04 |
| nether | 7 | 4.81 | 4.05 | 1.86 | 1.32 |
| **lovable** | **8** | **8.26** | **5.41** | **1.97** | **1.96** |

*Note. Words in bold were used as CSs during discrimination, the rest of the stimuli were Generalization Stimuli tested during test. All seven stimuli were tested during test.*

**Supplementary Table 9**: Concrete Noun Valence-Variable

| **Word** | **Level** | **Valence** | **Arousal** | **Concreteness** | **Log Frequency** |
| --- | --- | --- | --- | --- | --- |
| **mugger** | **2** | **1.90** | **5.67** | **4.27** | **1.69** |
| wounds | 3 | 2.95 | 4.00 | 4.19 | 2.85 |
| servant | 4 | 4.00 | 3.77 | 4.64 | 2.79 |
| platform | 5 | 5.00 | 4.18 | 4.48 | 2.49 |
| medicine | 6 | 5.90 | 4.00 | 4.79 | 3.24 |
| concert | 7 | 7.00 | 5.17 | 4.35 | 2.95 |
| **laughter** | **8** | **8.26** | **5.41** | **1.97** | **1.96** |

*Note. Words in bold were used as CSs during discrimination, the rest of the stimuli were Generalization Stimuli tested during test. All seven stimuli were tested during test.*

**Supplementary Table 10:** Concrete Noun Valence-Fixed

| **Word** | **Level** | **Valence** | **Arousal** | **Concreteness** | **Log Frequency** |
| --- | --- | --- | --- | --- | --- |
| **mugger** | **2** | **1.90** | **5.67** | **4.27** | **1.69** |
| headline | 3 | 5.00 | 4.14 | 4.04 | 2.12 |
| machine | 4 | 5.00 | 4.39 | 4.25 | 3.55 |
| platform | 5 | 5.00 | 4.18 | 4.48 | 2.49 |
| pulley | 6 | 5.00 | 4.00 | 4.74 | 1.36 |
| cauldron | 7 | 4.91 | 4.41 | 4.61 | 1.39 |
| **laughter** | **8** | **8.26** | **5.41** | **1.97** | **1.96** |

*Note. Words in bold were used as CSs during discrimination, the rest of the stimuli were Generalization Stimuli tested during test. All seven stimuli were tested during test.*

**Supplementary Table 11:** Concrete Adjective Valence-Variable

| **Word** | **Level** | **Valence** | **Arousal** | **Concreteness** | **Log Frequency** |
| --- | --- | --- | --- | --- | --- |
| **killer** | **2** | **1.74** | **7.05** | **3.31** | **3.50** |
| bloody | 3 | 2.86 | 5.76 | 4.08 | 3.29 |
| wrinkled | 4 | 3.74 | 3.80 | 4.29 | 1.93 |
| curling | 5 | 5.00 | 4.43 | 4.04 | 1.69 |
| blonde | 6 | 6.00 | 5.75 | 4.52 | 2.85 |
| bright | 7 | 6.84 | 5.00 | 3.92 | 3.35 |
| **wildlife** | **8** | **7.75** | **5.67** | **4.26** | **1.99** |

*Note. Words in bold were used as CSs during discrimination, the rest of the stimuli were Generalization Stimuli tested during test. All seven stimuli were tested during test.*

**Supplementary Table 12:** Concrete Adjective Valence-Fixed

| **Word** | **Level** | **Valence** | **Arousal** | **Concreteness** | **Log Frequency** |
| --- | --- | --- | --- | --- | --- |
| **killer** | **2** | **1.74** | **7.05** | **3.31** | **3.50** |
| concrete | 3 | 4.82 | 3.63 | 4.59 | 2.57 |
| staple | 4 | 5.00 | 4.48 | 4.34 | 1.76 |
| curling | 5 | 5.00 | 4.43 | 4.04 | 1.69 |
| striped | 6 | 4.81 | 4.38 | 4.04 | 1.94 |
| vertical | 7 | 5.00 | 4.67 | 3.48 | 2.15 |
| **wildlife** | **8** | **7.75** | **5.67** | **4.26** | **1.99** |

*Note. Words in bold were used as CSs during discrimination, the rest of the stimuli were Generalization Stimuli tested during test. All seven stimuli were tested during test.*

**2. Training**

A mixed ANOVA with 2 (Group: Valence-Variable vs Valence-Fixed) x 2 (CS: CS+ vs CS-) was used to analyse the last training trial. It revealed the expected effect of CS, *F*(1,135) = 467, *p* < .001, *η^2^_p_*= .77, 95% CI [.71, .82]. No other effect was significant, largest F for the group factor, *F*(1,135) = 1.03, *p* = .312, suggesting that the discrimination was successful without differences between groups.

**Supplementary Figure 4:** Training of Experiment 2

*Note. Error bars represent 95% CI.*

**3. Supplementary Expectancy Test**

*CS+ Valence:*

A mixed ANOVA with 2 (Group: Valence-Variable vs Valence-Fixed) x 7 (Level:1-7) x 2 (Cs+Valence: Positive vs. Negative) revealed a triple interaction, *F*(5.24, 697.21) = 5.37, *p* < .001, *η^2^_p_*= .04, 95% CI [.01, .06]. As Supplementary Figure 5 shows, the linear gradient for Group Valence-Variable in the conditioning with negative valence (Figure S5A) was more pronounced than in the case of conditioning with positive valence (Figure S5B). Indeed, analyses of the slope of the 5GS, showed differences between Variable and Fixed groups in the conditioning with negative valence (Figure S5C), *t*(70) = 4.23, *p* < .001, *d*= 0.99, but not in the conditioning with positive valence(Figure S5D), *t*(63) =0.99, *p* = .325. In the case of conditioning with positive valence, variable and fixed groups showed a similar pattern of responses, without a gradient. Indeed, in the case of positive conditioning the slope in group Valence Variable was not different from zero, *t*(34) =1.78, *p* = .083.

**Supplementary Figure 5**: Expectancy Test as a function of CS+Valence

*Note. Panel A represents the expectancy test when the CS+ has negative valence and Panel B when the CS+ has positive valence (black symbols for group Valence Variable and white symbol for group Valence Fixed). Panel C represents the violin plot of the slope when the CS+ has negative valence Panel D when the CS has positive valence. Black lines represent the median and red lines the quartiles. Error bars are the 95% CIs.*

*Words Attributes:*

A Mixed 2 (Group: Valence-Variable vs Valence-Fixed) x 7 Level x 2 (Concreteness: Abstract vs Negative) x 2 (Type of Word: Noun vs Adjetive) ANOVA revealed a interaction Concreteness x Level, *F*(4.72, 637.18) = 4.41, *p* = .002, *η^2^_p_*= .03, 95% CI [.01, .06], but not interaction between TypeWord x Level, *F*(4.72, 637.18) = 1.62, p = .155, nor triple interaction F < 1. As Supplementary Figure 6 shows, the linear gradient for Group Valence-Variable in the abstract condition (Figure S6A) was more evident than in the case of concrete condition (Figure S6B). Indeed, analyses of the slope of the 5GS, showed differences between Variable and Fixed groups in the abstract condition (Figure S6C), *t*(67) = 4.90, *p* < .001, *d*= 1.18, but not in the concrete condition (Figure S6D), *t(*66) = 0.83, *p* = .409.

**Supplementary Figure 6**: Expectancy Test Words Attributes

*Note. Panel A represents the expectancy test when the CS+ has negative valence and Panel B when the CS+ has positive valence (black symbols for group Valence Variable and white symbol for group Valence Fixed). Panel C represents the violin plot of the slope when the CS+ has negative valence Panel D when the CS has positive valence. Black lines represent the median and red lines the quartiles. Error bars are the 95% CIs.*
